# Portability of IMRT QA between matched linear accelerators

**DOI:** 10.1002/acm2.14492

**Published:** 2024-09-09

**Authors:** Brendan Barraclough, Zacariah E. Labby, Sean P. Frigo

**Affiliations:** ^1^ Department of Radiation Oncology Duke University Medical Center Durham North Carolina USA; ^2^ Department of Human Oncology University of Wisconsin ‐ Madison Madison Wisconsin USA

**Keywords:** beam match, IMRT QA, matched linacs, patient specific QA, portability

## Abstract

**Purpose:**

To determine if patient‐specific IMRT quality assurance can be measured on any matched treatment delivery system (TDS) for patient treatment delivery on another.

**Methods:**

Three VMAT plans of varying complexity were created for each available energy for head and neck, SBRT lung, and right chestwall anatomical sites. Each plan was delivered on three matched Varian TrueBeam TDSs to the same Scandidos Delta^4^ Phantom+ diode array with only energy‐specific device calibrations. Dose distributions were corrected for TDS output and then compared to TPS calculations using gamma analysis. Round‐robin comparisons between measurements from each TDS were also performed using point‐by‐point dose difference, median dose difference, and the percent of point dose differences within 2% of the mean metrics.

**Results:**

All plans had more than 95% of points passing a gamma analysis using 3%/3 mm criteria with global normalization and a 20% threshold when comparing measurements to calculations. The tightest gamma analysis criteria where a plan still passed > 95% were similar across delivery systems—within 0.5%/0.5 mm for all but three plan/energy combinations. Median dose deviations in measurement‐to‐measurement comparisons were within 0.7% and 1.0% for global and local normalization, respectively. More than 90% of the point differences were within 2%.

**Conclusion:**

A set of plans spanning available energies and complexity levels were delivered by three matched TDSs. Comparisons to calculations and between measurements showed dose distributions delivered by each TDS using the same DICOM RT‐plan file meet tolerances much smaller than typical clinical IMRT QA criteria. This demonstrates each TDS is modeled to a similar accuracy by a common class (shared) beam model. Additionally, it demonstrates that dose distributions from one TDS show small differences in median dose to the others. This is an important validation component of the common beam model approach, allowing for operational improvements in the clinic.

## 1 INTRODUCTION

Modern medical linear accelerators are produced with sufficient uniformity that some clinics have found they can use a common beam model for multiple treatment delivery systems (TDSs).^1^, ^2^, ^3^, ^4^ Specifically, the authors’ clinic operates three TrueBeam medical linear accelerators (Varian Medical Systems, USA). Each of these TDSs is in three physically distinct locations; travel between them takes approximately 30−60 min, though patients are routinely transferred between the three systems.

The existing TrueBeam TDSs are considered matched for the purposes of treatment planning—there is a single beam model class in the treatment planning system (TPS) for all three. This is made possible by establishing at time of acceptance and commissioning that each treatment delivery system adheres to the clinics’ machine performance specification. This ensures machine output, percent depth doses, and profiles all lie within a specified range, and other system quality management is matched between TDSs. This allows the use of a single model class in the TPS. Additionally, any new TDSs of the same design not initially meeting the machine performance specification could be tuned to match the others and fall into the same model.

However, previous work has shown that matching secondary machines to a primary reference machine does not guarantee the new machines will match the TPS.^5^ Therefore additional testing needs to be done, e.g., according to MPPG 5, to further verify the dosimetric match between each machine and the TPS in these clincs.^6^, ^7^, ^8^

A conservative approach is to perform patient‐specific IMRT quality assurance (QA) separately for each TDS used to deliver a given plan. If a patient is transferred from one delivery system to another, a new QA measurement will be performed on the new delivery system. Even if all matched delivery systems were in the same building, performing QA on each machine causes duplication of effort. With the machines being physically separated, there are additional requirements for physics measurements at the three sites beyond what may be otherwise required. Finally, a given machine will sometimes be available during the treatment day; currently, this time can be used for performing QA for plans to be delivered at that machine, but not for plans on other machines that also require QA. Another overly constrained situation is when a machine is down until late at night, making QA difficult to complete prior to a new start the following day.

The purpose of this work was to gather and analyze data to determine the portability of patient‐specific IMRT QA between delivery systems. Specifically, this work sought to determine if a plan's QA measurement can be safely performed at any matched delivery system, regardless of which would be used to treat the patient. The goal was to improve operational efficiency and robustness. If QA can be delivered at any available TDS, all measurements can be taken in a single session with less concern for a single late or down machine. Establishing plan and measurement portability makes it possible that less physical presence from the physics team will be required at a given clinic site to provide the same level of quality coverage at the multiple locations. Finally, measurements could also be taken on a delivery system that is available during the treatment day, reducing the time a physicist needs to be in the clinic after hours.

## 2 MATERIALS AND METHODS

### 2.1 Equipment

This work used a Delta^4^ Phantom+ (Scandidos, Sweden) to measure delivered dose distributions. This device has two orthogonal detector boards with a total of 1069 diodes embedded in a cylinder of PMMA. Detectors are spaced 5 mm apart in the central region of each board and 10 mm in the remainder of the 20 × 20 cm^2^ detection region. An A1SL ion chamber (Standard Imaging, USA) was used with a stack of solid water and a Unidos electrometer (PTW, Germany) for daily TDS output measurement (and subsequent correction). The A1SL is an ionization chamber of 0.06 cm^3^ active volume that meets the specifications of the AAPM's TG‐51 addendum for reference‐class ion chambers for MV beam dosimetry.^9^

There were three TrueBeams investigated in this work, referred to here as TB1, TB2, and TB3. All three have the Millennium 120 MLC with 40 central leaves (0.5 cm width) and 20 outer leaves (1 cm width). TB2 and TB3 had five photon energies—6 MV flattened (6X), 6 MV flattening‐filter‐free (6FFF), 10 MV flattened (10X), 10 MV flattening‐filter‐free (10FFF), and 15 MV flattened. TB1 had 6X, 6FFF, 10X, and 10FFF photon beam energies. These three delivery systems are already considered matched for all available energies—one beam model was validated for all three delivery systems.

All three TrueBeams meet the same performance specifications as part of routine QA (daily, monthly, and yearly). To ascertain MLC positioning, mean MLC positioning offset results from the Machine Performance Check (MPC) routine were analyzed to identify any potential influence on the delivered dose to the Delta^4^ Phantom+.

Test plans were created using RayStation version 7.0.0.19 (RaySearch, Sweden) as the TPS. Dose calculations used a collapsed‐cone convolution/superposition algorithm and a dose grid spacing of 2 mm. Plans were optimized using standard clinical planning techniques.

### 2.2 Daily output correction

Absolute output was obtained once through a reference dosimetry measurement in a water tank acquired using the protocol detailed in the report from AAPM's TG‐51.^10^ Relative output was then measured using the A1SL chamber and a stack of solid water. A transfer coefficient was obtained to relate the relative output measurement in solid water to the absolute output measurement in the water tank. Relative output was measured for each TDS immediately prior to the Delta4+ measurements. The relative output correction factor and the correction for absolute dose deviation from the TPS under calibration conditions were combined to create “output correction factors” to correct for any output variation between delivery systems.

### 2.3 MLC positioning

The resulting dose distribution from a modulated treatment plan is dependent on the specific MLC positioning properties of the TDS, as well as the characteristics of the leaves themselves (e.g., rounded leaf‐end effects for TrueBeam MLCs). Tuning of the MLC leaf end properties for a beam model in a TPS is a common practice for c‐arm TDSs. Discrepancies in MLC positioning characteristics between TDSs (e.g., if the three TDSs had different calibrated MLC “gap” parameters in the service configuration for each machine) could result in the same radiation treatment plan delivery instructions producing different delivered dose distributions per TDS. The variation in MLC positioning between TDSs was determined from TrueBeam MPC results, specifically the mean offset position for the innermost leaves. Representative MPC values were taken from inspection of the graphical display over the time period of Delta4 Phantom+ measurements. From the offset values for each MLC bank (A, B), the spread and average were calculated.

### 2.4 Test plans

Three plans were selected for this work. They were chosen based on the need to test a range of target size and complexity that would cover the range of patient treatments delivered on these machines. The first was for a right‐side head and neck site (referred to here as NecR_VMA) with a target size of approximately 18 cm x 7 cm. The prescription dose 1.8 Gy per fraction with a simultaneous integrated boost on a smaller volume to 2 Gy per fraction for a total dose of 60 and 66 Gy. It is highly modulated with a modulation complexity score (MCS) score of 0.3–0.4 and irregularity of 6.2–9.2 (depending on energy).^11^, ^12^

The second was for a left‐sided lung SBRT site (referred to as LunL_SBR). The target size was approximately 4 cm x 4 cm with MCS of 0.2–0.3 and irregularity of 2.2–2.5. The prescription dose was 10 Gy per fraction. Due to the small size and high‐dose gradient, this plan was measured with a “high resolution” technique for the diode phantom, where two measurements are acquired with a longitudinal position shift equal to half the distance between the central diodes of the diode array (2.5 mm). These measurements are then merged to have a final measurement spacing in the IEC‐Y‐direction of 2.5 mm in the central region and 5 mm in the outer region.

Finally, a large right‐sided chestwall plan (referred to as ChwR_VMA) was measured. The MCS for this plan was 0.1–0.2 and irregularity ranged from 8.7 to 11.6. The prescription dose was 1.8 Gy per fraction. The target in this plan was large enough to necessitate an “extended field” measurement technique. This involves merging two measurements separated by a large shift in the y‐direction to encompass the entire length of the target.

Each plan was selected based on a clinically approved treatment plan used for patient care. All were VMAT plans with arc number and geometry based on standard patient care templates. Then, using the same optimization objectives and doses on the same patient geometry, the plan was reoptimized using the other available energies. To be clear, the plans reoptimized using different energies were not used for patient care, but they were optimized using the same clinical goals, and the final dose distributions thus approached those of the original clinical plans.

### 2.5 Delivered dose distribution measurement

The Delta4 Phantom+ was setup by visually aligning the device's marks to the in‐room lasers. When an initial rough alignment was found, further corrections were made using a dosimetric method using a simple, square‐field arc of 360 degrees delivered to the phantom. Given a measured and calculated dose for this delivery, the ScandiDos Delta4 software is able to compute the phantom shifts needed for an ideal alignment. These shifts were < 1 mm and were applied by shifting the couch. This adjustment was repeated until calculated shifts were no greater than 0.1 mm. In this way, the Delta4 Phantom+ was aligned to the beam isocenter.

Each of the plans were delivered for the three test sites described previously for each beam energy (6X, 6FFF, 10X, 10FFF, and 15X) for a total of 15 plans. This was done on each machine, with the exception of the 15X plans at TB1, as 15X photons are not available on that machine. The temperature was measured on the outside surface of the Delta4 Phantom+ at regular intervals to correct for variation in diode response. The time between temperature readings was small enough that consecutive measurements never differed by more than 0.3°C. The temperature was interpolated for plans that did not have their own distinct reading. The Delta4 software has a feature that allows for input of a scaling factor that will directly rescale the measured absolute dose. The daily output correction factors as described in section 2.B were applied using this feature.

Gamma analyses between the 3D composite measured data and the calculated data from the TPS were performed in the Delta4 software. One is able to determine passing rates for a wide range of criteria—from 0.5% to 5% and 0.5 to 5 mm in 0.5% and 0.5 mm increments, respectively. Only detectors in the dose range of 20%–500% of the prescription were included. Each gamma analysis was performed twice—once with the dose deviation normalized globally and again with dose deviation normalized locally.

Additionally, the measurements were compared between the three machines. This was performed by exporting the absolute dose values for the individual detectors. A dose difference comparison was then performed between measurements from each of the three machines in a round‐robin manner so that measurements from each machine was compared to each of the others. Only diodes measuring greater than 20% of the plan's prescribed dose were included in the analysis. The dose differences were normalized using two methods. The first was a global normalization, where the differences were normalized to the plan's prescribed dose:

$$Dose\;Deviation_{Global}=\left(M_{X}-M_{Y}\right)/Dose_{Rx}$$

where X and Y are the TDSs being compared.

The other method was a local normalization, where the difference between each pair of detectors was normalized to the reading from one of the diodes:

$$Dose\;Deviation_{Relative}=\left(M_{X}-M_{Y}\right)\;/\;M_{Y}$$

Finally, a normalized median dose deviation was determined for each normalization method.

## 3 RESULTS

### 3.1 MLC positioning

An analysis of the mean offset values is summarized in Table 1. Mean offsets for each bank of leaves for each delivery system are all within 0.2 mm. For Bank A of each machine, agreement is within 0.1 mm. The largest discrepancy for Bank B was between TB2 and TB3 at 0.17 mm. The rest were 0.1 mm or less.

**TABLE 1:** MPC mean offset value results.

Position (mm)				
Delivery system	Bank A	Bank B	Middle	Spread
TB1	−0.31	0.18	−0.07	0.49
TB2	−0.26	0.28	0.01	0.54
TB3	−0.32	0.11	−0.11	0.43

### 3.2 Gamma analysis

Gamma analyses were performed on each plan at the clinically used 3%/3 mm criteria with global normalization and a 20% threshold. These criteria were developed before the release of the report from the AAPM's TG‐218, which recommends using 3%/2 mm criteria with global normalization and a 10% threshold.^13^ For the criteria used in this work, greater than 95% of points in each plan had a gamma value less than one for all three plans at all five energies on each of the three machines. The lowest rate for this test was 99.0% for the 15X BreR_VMA plan on TB3, and most plans were at 100%. When a local normalization was applied, two plans had fewer than 95% of points with gamma values less than one. Namely, the 15X BreR_VMA plan on TB2 and the 15X BreR_VMA plan on TB3 had passing rates of 90.2% and 84.9%, respectively (It should be noted that this clinic does not use IMRT or VMAT at 15X beam energies clinically; the 15X test plans were only measured to assess plan and measurement portability across the full range of TDS capabilities.)

A broader analysis was also done with a wide range of gamma criteria. Table 2 presents a selection from the NecR_VMA plan delivered at 6X on all three machines. These are the passing rates when the dose deviation is normalized globally. Tables 3 and 4 give the passing rate for the smallest symmetrical criteria (criteria where the numerical value of the percent difference and distance‐to‐agreement were equal) that passed the gamma analysis at a rate of 95% or higher. Generally, the gamma analysis of a measurement on one machine was very similar to the analysis of a measurement on another machine for the same plan and energy. For each plan and at each energy, the smallest symmetrical criteria for which a passing result was achieved for each machine was within 0.5% and 0.5 mm. There were only two exceptions. One was the 6X version of the LunL_SBR plan with global normalization (0.5%/0.5 mm for TB1, 1.5%/1.5 mm for TB3). The second was the 10X version of the BreR_VMA plan with global normalization (1.5%/1.5 mm for TB1, 2.5%/2.5 mm for TB3).

**TABLE 2:** Gamma index passing rates for various criteria for the NecR_VMA 6X plan on each machine using a global dose deviation normalization. If 95% of points pass, the rate is highlighted in green. If < 95% of points pass, the rate is highlighted in red.

a. Passing rates for TB1.						
Dist dev (mm)	Gamma index pass rates (%)					
3	96.3	98.6	100	100	100	100
2.5	93.6	97.3	99.9	100	100	100
2	88.2	96.4	99.9	100	100	100
1.5	78.8	92.5	99.4	100	100	100
1	65.8	86.8	97.9	100	100	100
0.5	48.7	77.9	94.5	99.6	100	100
Dose dev (%)	0.5	1	1.5	2	2.5	3

b. Passing rates for TB2.						
Dist dev (mm)	Gamma index pass rates (%)					
3	97.1	98.9	99.9	100	100	100
2.5	95	97.9	99.9	100	100	100
2	92.6	96.5	99.9	100	100	100
1.5	84.8	94.7	99.6	100	100	100
1	74.9	90.8	99.3	100	100	100
0.5	61.7	83.8	97.3	99.9	100	100
Dose dev (%)	0.5	1	1.5	2	2.5	3

c. Passing rates for TB3.						
Dist dev (mm)	Gamma index pass rates (%)					
3	93.6	98.3	99.9	100	100	100
2.5	89.6	96.9	99.5	99.9	100	100
2	84.6	94.6	99.4	99.9	100	100
1.5	76.6	90	98.8	99.9	100	100
1	65.4	84.3	97	99.9	100	100
0.5	46.4	73.3	91.4	97.3	98.7	99.5
Dose dev (%)	0.5	1	1.5	2	2.5	3

**TABLE 3:** The tightest criteria where the gamma analysis with a global dose deviation normalization passed as well as the passing rate at that criterion for each plan, energy, and machine.

	NecR_VMA, Global normalization									
	6X		6FFF		10X		10FFF		15X	
TrueBeam	Criteria (% & mm)	Pass rate (%)	Criteria (% & mm)	Pass rate (%)	Criteria (% & mm)	Pass rate (%)	Criteria (% & mm)	Pass rate (%)	Criteria (% & mm)	Pass rate (%)
TB1	1.5	99.4	1.5	96.2	1.0	95.5	1.5	98.8	—	—
TB2	1.5	99.6	1.5	97.2	1.5	99.9	1.5	98.6	2.0	97.7
TB3	1.5	98.8	1.5	95.6	1.5	98.2	2.0	98.5	2.0	98.3

	LunL_SBR, Global normalization									
	6X		6FFF		10X		10FFF		15X	
TrueBeam	Criteria (% & mm)	Pass rate (%)	Criteria (% & mm)	Pass rate (%)	Criteria (% & mm)	Pass rate (%)	Criteria (% & mm)	Pass rate (%)	Criteria (% & mm)	Pass rate (%)
TB1	0.5	97.3	1.0	96.7	1.0	100.0	1.0	100.0	—	—
TB2	1.0	100.0	1.0	96.7	1.0	100.0	1.0	100.0	1.5	100.0
TB3	1.5	96.5	1.0	98.6	1.0	100.0	1.0	100.0	1.5	100.0

	BreR_VMA, Global normalization									
	6X		6FFF		10X		10FFF		15X	
TrueBeam	Criteria (% & mm)	Pass rate (%)	Criteria (% & mm)	Pass rate (%)	Criteria (% & mm)	Pass rate (%)	Criteria (% & mm)	Pass rate (%)	Criteria (% & mm)	Pass rate (%)
TB1	2.0	98.1	2.0	98.7	1.5	95.9	2.0	97.2	—	—
TB2	2.0	98.0	2.0	96.9	2.0	97.2	2.5	99.2	3.0	99.7
TB3	2.0	96.8	2.0	95.2	2.5	99.5	2.5	97.9	3.0	99.0

**TABLE 4:** The tightest criteria where the gamma analysis with a local dose deviation normalization passed as well as the passing rate at that criterion for each plan, energy, and machine.

	NecR_VMA, Local normalization									
	6X		6FFF		10X		10FFF		15X	
TrueBeam	Criteria (% & mm)	Pass rate (%)	Criteria (% & mm)	Passing rate (%)	Criteria (% & mm)	Passing rate (%)	Criteria (% & mm)	Passing rate (%)	Criteria (% & mm)	Passing rate (%)
TB1	2.5	97.3	2.5	95.2	2.0	98.8	2.0	99.1	—	—
TB2	2.5	97.9	2.5	96.5	2.0	97.3	2.0	98.2	2.5	95.7
TB3	2.5	97.0	2.5	95.4	2.0	96.0	2.5	98.5	2.5	96.5

	LunL_SBR, Local normalization									
	6X		6FFF		10X		10FFF		15X	
TrueBeam	Criteria (% & mm)	Pass rate (%)	Criteria (% & mm)	Passing rate (%)	Criteria (% & mm)	Passing rate (%)	Criteria (% & mm)	Passing rate (%)	Criteria (% & mm)	Passing rate (%)
TB1	1.0	99.7	1.0	96.7	1.0	99.5	1.0	98.3	—	—
TB2	1.0	97.8	1.0	96.5	1.0	99.7	1.0	98.1	1.5	96.9
TB3	1.5	96.5	1.0	98.6	1.0	100.0	1.0	100.0	1.5	100.0

	BreR_VMA, Local normalization									
	6X		6FFF		10X		10FFF		15X	
TrueBeam	Criteria (% & mm)	Pass Rate (%)	Criteria (% & mm)	Passing Rate (%)	Criteria (% & mm)	Passing Rate (%)	Criteria (% & mm)	Passing Rate (%)	Criteria (% & mm)	Passing Rate (%)
TB1	2.5	97.2	2.5	95.2	2.0	95.4	2.5	95.6	—	—
TB2	2.5	95.0	3.0	98.2	2.5	95.6	3.0	96.7	3.5	97.6
TB3	3.0	97.6	3.0	97.1	3.0	98.1	3.5	97.9	3.5	95.0

### 3.3 Dose deviation

Comparisons between measured doses from the three TDSs were also conducted. Tables 5 and 6 show the median percent dose deviation and the number of points within ± 2% of the median for this point‐by‐point comparison for each plan, energy, and machine combination. Table 5 displays results when the deviations were calculated as a percentage of the prescribed dose for that plan, namely, global normalization. Table 6 displays the results when the deviations were a percentage of the dose at each detector, namely, local normalization. Over all combinations of plan, energy, and machine, the largest median dose deviation was 0.7% of the plan's prescribed dose when global normalization was used. That result was the median of the dose deviations between the measurements from TB3 and TB1 for the BreR_VMA (TPL_194) plan at 10X. When a local normalization was performed instead, the greatest median dose deviation was 1.0%, again for the measurements from TB3 and TB1 for the BreR_VMA (TPL_194) plan at 10X. In the global normalization analysis, for each comparison, 86% or more of all dose deviations (for diodes measuring more than 20% of the prescription) were less than ± 2%. In most cases, 95% were within ± 2%. Generally, for this metric, TB3 and TB1 had the worst agreement, but not in every case. For the local normalization analysis, the values were lower, as expected. The LunL_SBR plan had the worst results across the board, with one comparison having 68% of deviations within ± 2%. However, for all combinations, more than 80% of deviations were within ± 3%.

**TABLE 5:** Median dose deviations as a percent of the prescription dose for that plan (global normalization). The median and the percent of deviations within 2% are given.

	Dose deviation (%)—global normalization																	
Median & Percent within 2% of median																		
Energy	6X						6FFF						10X					
Plan	NecR		LunL		BreR		NecR		LunL		BreR		NecR		LunL		BreR	
TB1‐TB2	0.23	100	−0.19	96	0.01	100	0.11	99	−0.25	89	−0.18	99	−0.20	99	−0.21	90	−0.43	99
TB3‐TB2	0.27	97	−0.13	89	0.22	99	0.35	97	0.09	96	0.22	99	0.20	98	0.07	93	0.22	99
TB3‐TB1	0.04	93	0.11	86	0.23	98	0.19	94	0.47	90	0.39	96	0.40	93	0.43	92	0.69	96

10FFF						15X					
NecR		LunL		BreR		NecR		LunL		BreR	
−0.10	98	−0.25	89	−0.30	98	—	—	—	—	—	—
0.26	98	−0.09	91	0.26	99	−0.26	92	−0.01	87	0.24	100
0.40	92	0.34	93	0.59	94	—	—	—	—	—	—

**TABLE 6:** Median dose deviations normalized to dose at each point of measurement (local normalization). The median and the percent of deviations within 2% are given.

	Dose deviation (%)—local normalization																	
Median & Percent within 2% of median																		
Energy	6X						6FFF						10X					
Plan	NecR		LunL		BreR		NecR		LunL		BreR		NecR		LunL		BreR	
TB1‐TB2	0.37	97	−0.54	80	0.01	95	0.20	93	−0.52	70	−0.26	93	−0.31	93	−0.39	70	−0.63	93
TB3‐TB2	0.44	87	−0.34	81	0.31	91	0.55	89	0.21	84	0.32	92	0.32	89	0.16	82	0.30	87
TB3‐TB1	0.05	82	0.29	69	0.35	86	0.32	83	0.83	74	0.60	84	0.63	80	0.75	75	0.96	81

10FFF						15X					
NecR		LunL		BreR		NecR		LunL		BreR	
−0.16	92	−0.51	68	−0.45	90	—	—	—	—	—	—
0.47	91	−0.22	74	0.36	92	−0.45	76	−0.02	71	0.29	93
0.66	77	0.75	79	0.83	80	—	—	—	—	—	—

**FIGURE 1:**
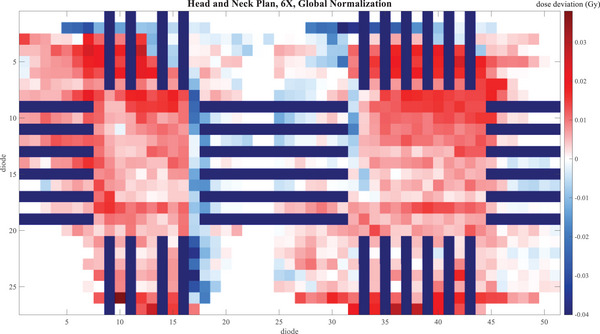
Dose deviation map for TB1 ‐ TB2 for the NecR_VMA plan with 6X beam energy for each diode in the Delta4 Phantom+. The dark blue lines are gaps in the diode array.

**FIGURE 2:**
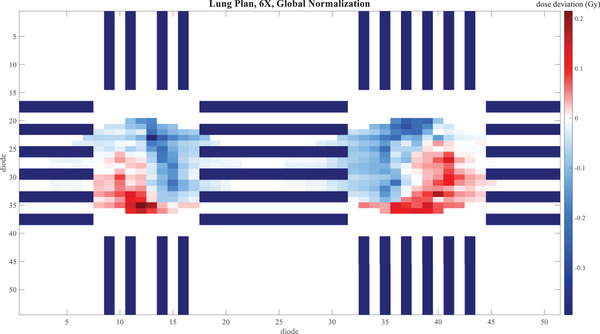
Dose deviation map for TB1 ‐ TB2 for the LunL_SBR plan with 6X beam energy for each diode in the Delta4 Phantom+. The dark lines are gaps in the diode array.

**FIGURE 3:**
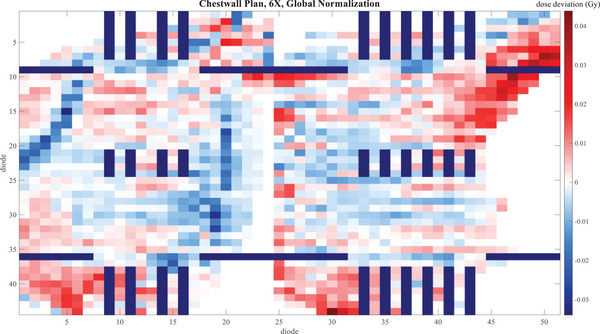
Dose deviation map for TB1 ‐ TB2 for the ChwR_VMA plan with 6X beam energy for each diode in the Delta4 Phantom+. The dark lines are gaps in the diode array.

Figures 1, 2, 3 give a visual representation of the dose deviation across the boards of the Delta4 Phantom+ for a small selection of the measurements—NecR_VMA, LunL_SBR, and ChwL at 6X, comparing TB3 to TB2. Only diodes measuring above the 20% threshold are shown. The dark lines, which are gaps in the measurement, vary from one plan to the next due to the different shifts in the merged measurements.

**FIGURE 4:**
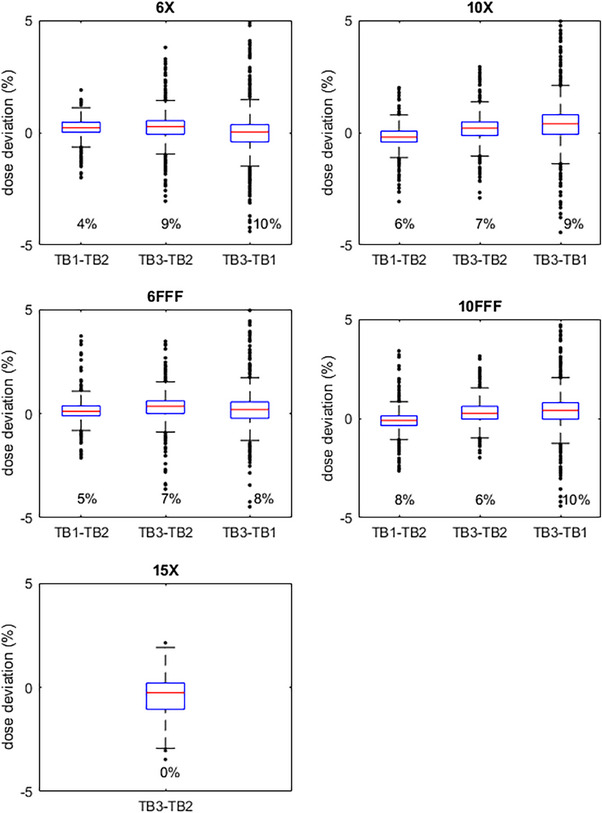
NecR_VMA: Whisker plots of dose deviations of each TDS‐to‐TDS comparison for each energy. Whisker lengths are 1.5x the interquartile range. Percent of points outside the whiskers also shown.

**FIGURE 5:**
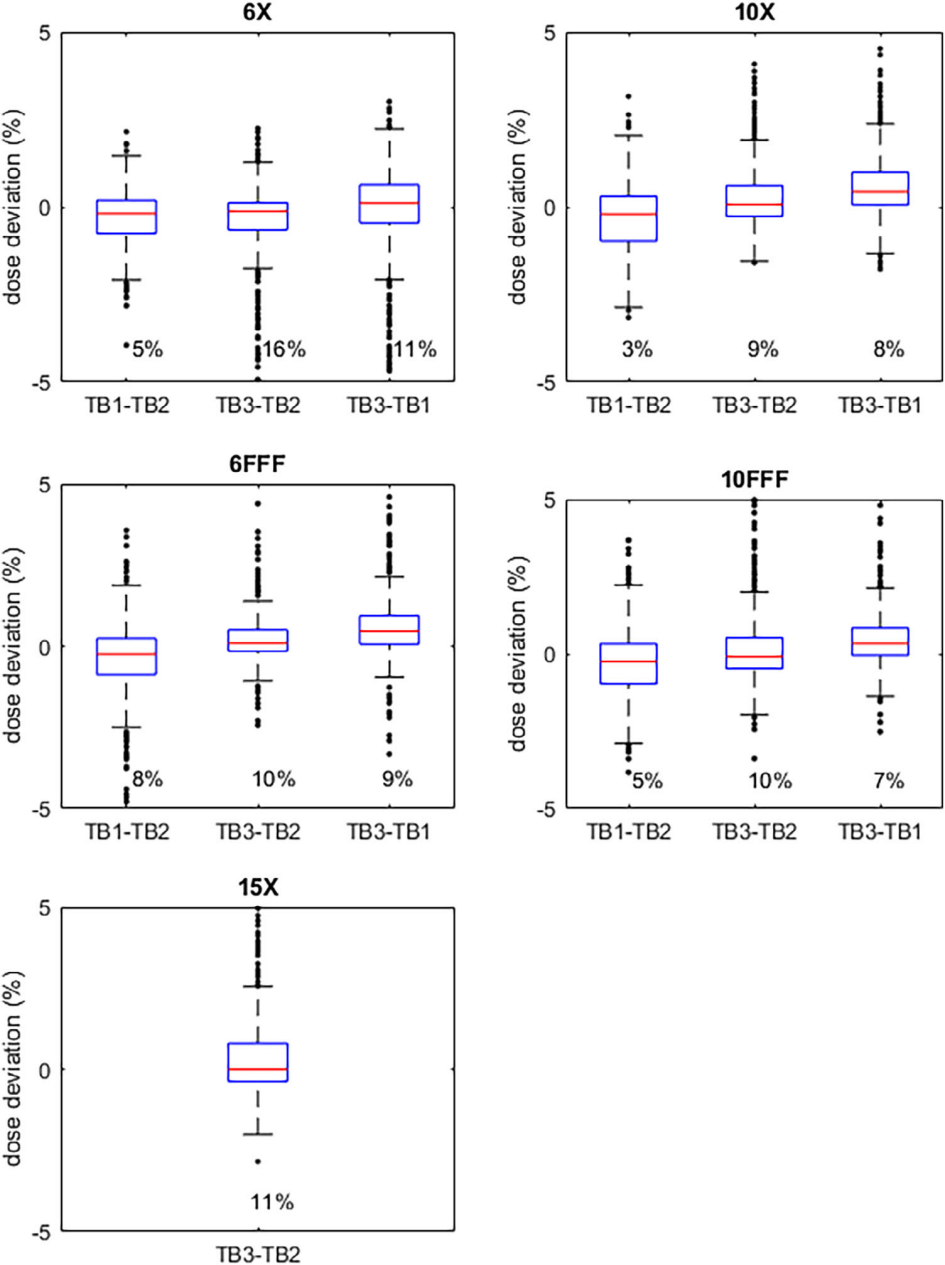
LunL_SBR: Whisker plots of dose deviations of each TDS‐to‐TDS comparison for each energy. Whisker lengths are 1.5x the interquartile range. Percent of points outside the whiskers also shown.

**FIGURE 6:**
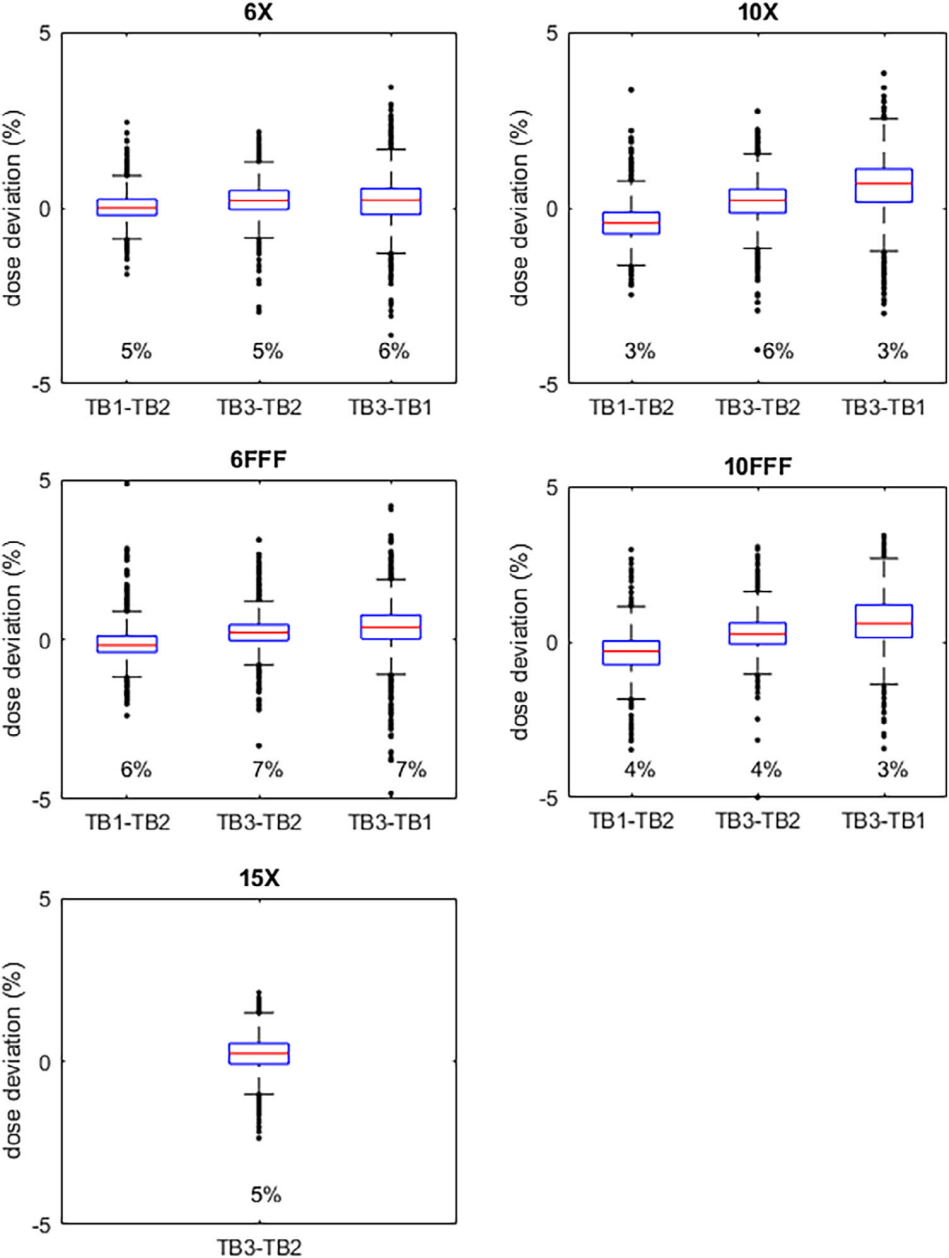
BreR_VMA: Whisker plots of dose deviations of each TDS‐to‐TDS comparison for each energy. Whisker lengths are 1.5x the interquartile range. Percent of points outside the whiskers also shown.

Additionally, whisker plots of dose deviations are shown in Figures 4, 5, 6. Each TDS‐to‐TDS comparison is shown for each energy and for all three plans. The whisker lengths are set to 1.5 times the interquartile range. Deviations outside of this range are shown on the plot as points, and their number as a percentage of the total number of points is displayed in the plot.

## 4 DISCUSSION AND CONCLUSION

The intent of this work was to determine how well the three TrueBeams in use in these clinics were matched in their ability to deliver VMAT plans of various target size and modulation level. While the full extent of the equivalence of these machines requires additional investigation, this initial goal was achieved.

There is already a degree of confidence in and reliance on the similarity of the treatment beams at each machine, based on initial acceptance testing and commissioning results and validation of our clinical beam model in our TPS as recommended in MPPG 5.^6^, ^7^ These two items are further underpinned by the consistent and similar MLC positioning in the MPC results. Such consistency increases the confidence of obtaining similar Delta4 Phantom+ measurement results. The more explicit goal of this work was to determine if patient‐specific IMRT QA could be performed at any machine rather than specifically at the machine on which it would be delivered to the patient.

Three general plans were used in the investigation, each planned with five energies. They spanned the range of small to large target size and had varying degrees of modulation. Each plan/energy combination was delivered on all machines, with the exception of 15X plans at TB1, which does not have a commissioned 15X beam. To isolate the differences in measured doses to differences solely in the machines to the greatest degree possible, steps were taken to reduce confounding factors in three specific ways.

The first was to use the same Delta4 Phantom+ for all measurements. In this way, the same Delta4 Phantom+ calibration was used and any variation in detector response between the different examples of the Delta4 Phantom+ in use in these clinics were removed. Second, the measurements were taken in as short of a time frame as reasonable. All measurements were gathered within approximately 2 weeks. This reduced any variation in the single Delta4 Phantom+’s detector response with accumulated dose or time. Finally, the daily output was measured at each machine. The correction factors found for output at the time of measurement were 1% or less for each TDS, but were a necessary component for building confidence in the results of this work. If output variations between TDSs were not removed, the results of this work would have been dependent on how recently the output of each TDS had been set. Removal of these three confounding factors enabled a more direct test of the beam model to a common TrueBeam configuration.

Additional work has been done to further demonstrate conformity between matched machines. Hansen and Frigo tested multiple candidate beam models and demonstrated matching between two machines.^14^ They hypothesized that the degree of matching seen on these machines could allow another institution to use the same beam model for similar machines. Frigo et al. then showed that a beam model shared between two institutions with TDSs that all met Varian's enhanced beam conformance criteria could achieve satisfactory results in beam model validation based on MPPG 5.^6^, ^15^ While Frigo et al. investigated aspects not addressed herein, a limitation of that former work was that only one beam energy was investigated (6 MV).

This new work tested all available energies at the institution, including flattened and flattening‐filter‐free beams. Additionally, this work was able to control additional variables, such as using the same QA device and device calibration to acquire all measurements. Furthermore, since all machines were maintained under the same machine QA program, procedures and equipment used for routine machine QA were identical, which is not the case in an interinstitutional study.

Despite all machines meeting the machine performance specifications, there are still aspects of the machines and their radiation beams that are not exactly matched. For example, according to Varian's beam conformance specifications, there should be point‐to‐point conformance of ±1% at 4 and 13 cm from the central axis for a 10 cm x 10 cm and 30 cm x 30 cm field, respectively.^16^ However, there could be variation within that specification from one machine to another, which was not analyzed for this study. Including a comparison of IMRT QA results such as done in this work could therefore be a valuable test during the initial commissioning of a new TDS in a clinic with an existing TDS of the same model. Besides comparing an IMRT dose distribution from the new TDSs to a TPS‐calculated dose, comparing this dose distribution to the existing machine(s) would further verify that complex, modulated plans are delivered uniformly across the entire “fleet.” This commissioning comparison could then be used as the baseline for additional tests performed during periodic QA to verify conformity on an ongoing basis.

Based on the gamma analysis results and the small median dose deviations described in this work across the spectrum of measurements, we conclude that IMRT QA can be performed on any of the three TrueBeams examined in this work regardless of which machine the patient will be treated on. All plans on all machines passed using not only clinical criteria but more restrictive tolerance levels as well.

Additionally, median dose deviations were all below 1%. With a global normalization, median dose deviations were no larger than 0.69%; most were considerably lower than this. When using a local normalization, where the median dose deviation is expected to be larger, the greatest median dose deviation was 0.96%—still under 1% while using a more demanding test. The percentages of diode‐by‐diode deviations within 2% with a global normalization were also encouraging, as they fell within the range that is typically seen in clinical IMRT QA when comparing a measurement to a TPS calculation. This compares favorably to previous work comparing measurements from plans delivered on several different models of Varian linear accelerator where it was concluded that the deviations were within clinical acceptability.^17^

Deviations within 2% with a local normalization were lower, as expected, especially for the LunL_SBR plan. However, a diode‐by‐diode dose deviation analysis is susceptible to even the slightest setup error. This issue is magnified for SBRT plans such as LunL_SBR due to the high‐dose gradients present in this type of plan. It is also not out of the ordinary to see larger dose deviations for smaller target SBRT plans than seen in typical IMRT plans. The whisker plots show that some individual points have larger deviations, but many of these are in high‐dose gradient regions, as seen in Figure 2, and this is the reason gamma analysis is widely favored over point‐by‐point dose deviations for analyzing plan QA measurements for clinical acceptability. It is reasonable to believe that similar results will be seen for other plans that fall within the spectrum of plans tested in this work.

## AUTHOR CONTRIBUTIONS

All authors contributed to the design of the study, analysis of the data, and review of the manuscript and figures. Brendan Barraclough performed the data acquisition.

## CONFLICT OF INTEREST STATEMENT

The authors have no relevant conflict of interest to disclose.
